# Toward Vasculature in Skeletal Muscle-on-a-Chip through Thermo-Responsive Sacrificial Templates

**DOI:** 10.3390/mi11100907

**Published:** 2020-09-30

**Authors:** Li Wan, James Flegle, Burak Ozdoganlar, Philip R. LeDuc

**Affiliations:** 1 Department of Mechanical Engineering, Carnegie Mellon University, 5000 Forbes Ave, Pittsburgh, PA 15213, USA; lwan1@andrew.cmu.edu (L.W.); ozdoganlar@cmu.edu (B.O.); 2 Department of Microbiology, University of Chicago, 5801 S Ellis Ave, Chicago, IL 60637, USA; jamesflegle6@uchicago.edu

**Keywords:** microfluidics, muscle-on-a-chip, sacrificial template

## Abstract

Developing new approaches for vascularizing synthetic tissue systems will have a tremendous impact in diverse areas. One area where this is particularly important is developing new skeletal muscle tissue systems, which could be utilized in physiological model studies and tissue regeneration. To develop vascularized approaches a microfluidic on-chip design for creating channels in polymer systems can be pursued. Current microfluidic tissue engineering methods include soft lithography, rapid prototyping, and cell printing; however, these have limitations such as having their scaffolding being inorganic, less desirable planar vasculature geometry, low fabrication efficiency, and limited resolution. Here we successfully developed a circular microfluidic channel embedded in a 3D extracellular matrix scaffolding with 3D myogenesis. We used a thermo-responsive polymer approach with micromilling-molding and designed a mixture of polyester wax and paraffin wax to fabricate the sacrificial template for microfluidic channel generation in the scaffolding. These findings will impact a number of fields including biomaterials, biomimetic structures, and personalized medicine in the future.

## 1. Introduction

Skeletal muscle tissue is critical in musculoskeletal systems especially with respect to force generation [1] as they contain myofibers with a high density of myotubes that are aligned in three-dimensional (3D) extracellular matrix (ECM) [2]. However, while this tissue is important for many functions, in vivo muscle tissues have limited regeneration abilities such as in injury and aging [1]. Thus, one direction with tremendous promise is synthesizing new muscle tissue. Biomimetic engineering muscle systems may be an effective approach to create in vivo muscle tissues that could be used in a diversity of areas including during surgery, and reconstruction. To create these tissues, one potential approach is to use skeletal muscle cell lines such as C2C12 cells, which have also been used in a variety of in vitro models including microfluidics [3,4] and are well studied in areas such as myogenesis and contraction [5,6,7]. 

Artificial tissue fabrication and ex vivo constructs have long been investigated in various fields. Functional tissue has previously been implanted in vivo to replace tissues like skin [8], cartilage [9], and bone [10]. One significant challenge in 3D designs of artificial tissue though is the limitations of the thickness due to nutrient and oxygen delivery. Similar to tumor necrosis, most thick tissues will have hypoxia as well as nutrient insufficiency when lacking vasculature due to limitations of diffusion. For in vivo tissue, the vasculature allows delivery across the length and depth of the tissues, which enables maintenance, growth, and development. Thus, a critical approach in engineering muscle tissue is to create vasculature within the 3D muscle tissues [11]. One approach to accomplish this vascularization is to apply microfluidic fabrication techniques. Features of these vasculatures would include appropriate vessel diameter range (for blood vessel, this range could vary from a few micrometers up to a few millimeters [12,13]), circular cross-section to mimic vessel systems, and a high throughput fabrication approach.

Existing techniques for microfluidic tissue engineering include soft lithography [14,15], rapid prototyping [16,17], and cell printing [18,19]. Soft lithography has been used for a number of years, yet its materials are mainly elastomer-based systems, which can be limited from a biocompatibility standpoint [20]. Thus using ECM based approaches could be extremely useful. In addition, microfluidic fabrication methods (e.g., soft lithography) usually generate planar 2D features, which are not geometrically similar to physiological vasculature. Gray-scale lithography [21] may provide some non-planar features, yet this is limited by the masks and resolution on these non-planar surfaces. Rapid prototyping [17] and cell printing [22] are additional methods that could provide 3D features, but they are also limited by resolution and can lack the ability for multiple cell seedings or co-cultures. The idea of applying sacrificial template with thermo-responsive materials such as wax [23,24,25] and gelatin [26] for microfluidic channel fabrication was also popular as it provided precision to replicate the morphology and the basis for 3D printing technique. 

**Figure 1:**
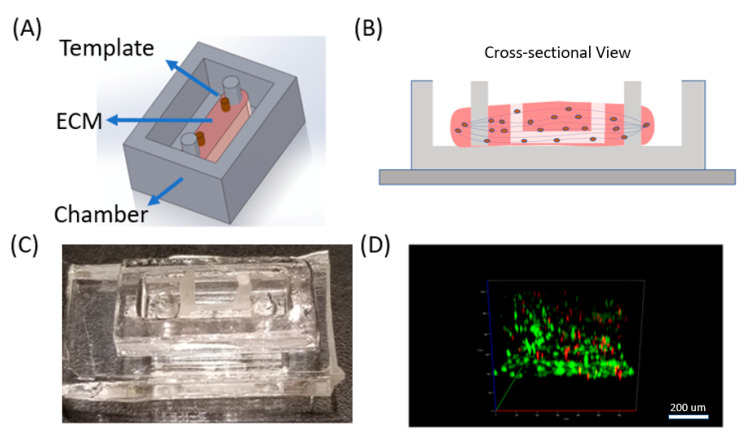
Toward vasculature in skeletal muscle-on-a-chip through themoresponsive sacrificial templates. (**A**) Schematic of general design of device with C2C12 cells cultured in a 3D collagen extracellular matrix (ECM) with the presence of a sacrificial template for vasculature formation. (**B**) Cross-sectional representation of the device. Myogenesis was induced, resulting in scaffold remodeling and parallel myotube formation, guided by double pillars in the chamber. The sacrificial template was removed leaving channels in the system. (**C**) Image of the device in the polydimethylsiloxane (PDMS) chambers with the wax template. (**D**) Live/dead staining confocal image of C2C12 cells embedded in 3D ECM in the device. Live cells were stained in green (Calcein AM) and dead cell in red (Ethidium homodimer).

Here we used a thermo-responsive polymer and micro-milling method [27,28] to fabricate sacrificial templates toward vasculature in 3D synthetic muscle ECM (Figure 1). We integrated an immortalized mouse myoblast cell line, C2C12, in a 3D collagen matrix (Figure 1A) and fabricated a vascularized layer within the ECM. For proof of principle, we fabricated a single circular channel, 500 um in diameter, with inlet and outlet for media perfusion purpose. We assessed cell viability, proliferation, and morphology with the presence of vasculature, and induced myogenesis of C2C12, with ECM remodeling (Figure 1B). We also seeded endothelial cells (HUVEC) into the vasculature to mimic blood vessels (Figure 1C). 

## 2. Materials and Methods

### 2.1. PDMS Chamber Fabrication with Pillars 

A polymethyl methacrylate (PMMA) chamber mold was fabricated by cutting 3 layers of PMMA (TAP Plastic, 2.4 cm in thickness) with a laser cutter (Epilog, Golden, CO, USA) and then adhering them together with optical adhesive (NOA 81, Norland products Inc., East Windsor, NJ, USA). Polydimethylsiloxane (PDMS, Ellsworth Adhesives, Germantown, WI, USA, 10:1 curing ratio) was poured into the mold, incubated at 60 °C for 2 h, and removed from the mold. PDMS chambers were rinsed in 70% ethanol under UV light for 2 h before being used for cell culture.

### 2.2. Wax Template Fabrication

20 g of polyester wax (AGL4184, Agar Scientific, Stansted, UK) and 2 g of paraffin wax (327212, Sigma-Aldrich, St. Louis, MO, USA) were stored in a glass bottle, heated for 20 min at 60 °C until they were completely melted, and then they were mixed together. The PDMS molds were fabricated with the same protocol reported previously [26,28]. In brief, the PMMA master mold was fabricated through micro-milling to generate parallel semicircular channels (4 mm in length). The master mold was then molded to produce positive replicas with polyvinyl siloxane (PVS, R-2364, Slipck Inc., Pomon, CA, USA), before being reverse molded into PDMS to recover the negative channels. Next, one PDMS mold was punched with needles (16G) at both ends of the channels to generate inlets and outlets. The PDMS molds were aligned and attached face-to-face.

The PDMS molds, a plastic syringe, and needle (20G) were all heated at 60 °C for 20 min. The wax mixture was also kept at 60 °C. Then the 5 mL of wax liquid, which was in the syringe, was injected into the PDMS mold. The mold was quickly frozen by placing it in a −20 °C freezer for 3 min. With a scalpel, we then removed the excessive wax at the inlet and outlet. Two dispensing needles with flat ends were used to carefully push the inlet and outlet to demold the wax template. The wax template was then rinsed in 70% ethanol for 2 h before using. It could be stored at room temperature for future use as well.

### 2.3. Muscle Cell Culture in 3D Collagen

C2C12 cells were obtained from ATCC and cultured in Dulbecco’s Modified Eagle’s high glucose medium (Thermofisher Scientific, Waltham, MA, USA) supplemented with 10% fetal bovine serum (FBS, Sigma-Aldrich) and 1% penicillin/streptomycin (Gibco). C2C12 cells cultured initially in a petri dish until they reached 70% confluency. At that point, they were trypsinized, resuspended in 593 mL of culture media and mixed thoroughly with 300 mL of high concentration collagen type I (Corning, Corning, NY, USA, 10 mg/mL), 100 mL of 10XPBS (Thermofisher Scientific) and 6.9 mL of 1N NaOH to obtain final collagen concentration of 3 mg/mL. Then the mixture was injected into the chamber with the wax templates, before being moved to the incubator (37 °C). The gelatin was removed with a syringe. One hundred microliters of media was added to the top of the device and the media was replenished every 12 h. For cells on the ECM surface, a blank collagen type I ECM was fabricated (using the same method discussed previously) and incubated at 37 °C to crosslink the collagen. Then 200 uL of culture media with C2C12 cells was pipetted on top of the ECM and incubated. To induce myogenesis, 10%FBS in culture media was replaced by 2% horse serum (HS).

### 2.4. Wax Template Removal from Collagen Scaffold

After 3 days of myogenesis and collagen remodeling, the wax template was removed. The device was transferred into the oven at 45 °C for 2 min so the wax quickly melted. The liquid wax could then be removed with a syringe (20G needle). 100 uL of PBS (warmed at 37 °C) was applied to wash the channel and remove wax residue at least twice.

### 2.5. Viability Test for Muscle Cell

For testing cell viability, the device was stained with 300 uL of CalAM (2 mM) and EthD-1 (4 mM) solution (L3224, Thermofisher Scientific) for 1 h to allow for diffusion into the collagen scaffold, and rinsed with PBS twice (10 min each). Then the device was immediately imaged using confocal microscopy (Axio Observer Z1 Microscope System, Zeiss, Oberkochen, Germany). Green fluorescent cells (EGFP positive) were counted as live cells and red cells were counted as dead cells. 

### 2.6. Endothelial Cell Culture in Microfluidic Channel

HUVEC cells were suspended in culture media (DMEM+10%FBS) at a density of 1 million cells/mL and then introduced into the channel inside the crosslinked ECM (C2C12 cells were pre-cultured in the ECM) and incubated for 4 h. The device was stored at 37 °C and to maintain viability of the HUVECs, fresh media was renewed in the channel every 12 h. 

## 3. Results and Discussion

### 3.1. Muscle Cells in Our ECM Based System and Vascular Cell Growth in Channels

**Figure 2:**
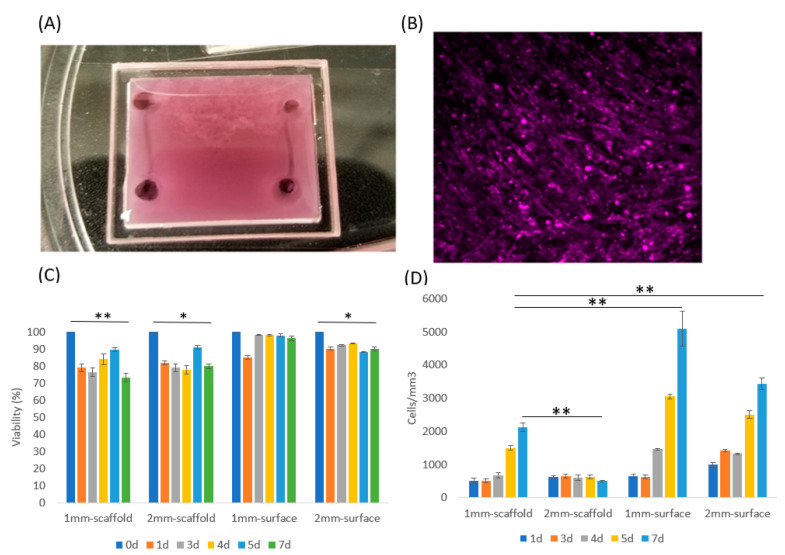
C2C12 culture in two and three dimensions. (**A**) The 3D ECM system with vascularized channels was made with a chamber made of a polymethyl methacrylate (PMMA) wall and coverslip. The chamber had dimensions of 17 × 19 × 2 mm. Two channels with D = 500 mm diameters were fabricated at both ends. (**B**) C2C12 cells were cultured on a 2D petri dish for 3 days with media and then the cells were stained with CellTracker DeepRed to examine cell viability. (**C**) Viability of 4 samples: Cells seeded in 1 mm thick collagen scaffold, 2 mm thick collagen scaffold, and cells cultured on the surface of 1 mm thick collagen and 2 mm thick collagen. Cell viability on 2.5D surface maintained higher than in 3D scaffold, according to Student T test. (**D**) The cell density of the 4 samples. Data were obtained by counting average cell numbers in each mm^3^ volume in 3D or 2.5D. Error bar: SD from 3 repeats. Student T test (one tail) was applied to obtain statistical significance. *, *p* < 0.05; **, *p* < 0.01.

The cells were characterized inside the 3D system to determine their structure and response. C2C12 cells are an immortalized mouse myoblast cell line, which proliferate well in standard culture conditions (DMEM with 10%FBS) and differentiate into myoblast under low serum culture condition (DMEM with 2%HS) [6]. After differentiation, myoblasts quickly can fuse into myotubes through myogenesis and remodel their ECM [29]. To determine C2C12 cell adaptation in our ECM based system, we first cultured C2C12 cells with DMEM+10%FBS in our device with 3 mg/mL collagen type I (Figure 2A). We fabricated two 500 um diameter channels in our ECM scaffolding and analyzed C2C12 response with a focus on differences relative to distances away from the vasculature. We tested 3 types of ECM conditions: (1) On the surface of a petri dish (hard 2D surface, Figure 2B), within the ECM scaffold (in 3D, Figure S1A,B), and on the surface of an ECM scaffold (soft 2.5D surface, Figure S1C,D). In addition, since tissue thickness could be a major factor that restricts cell proliferation and viability [30], we used a second approach, which examined ECM at controlled thicknesses of 1 mm and 2 mm (1 mm is considered an upper limit for tissue thickness with respect to necrosis [31]). 

To address the effects of thickness, we investigated C2C12 grown inside collagen type I, with 1 mm and 2 mm thicknesses (Figure S1A,B). Simultaneously, we wanted to understand the response on surfaces as that is more conventional for these systems, so we cultured C2C12 on collagen type I surface 2.5D with 1 mm and 2 mm ECM thickness (Figure S1C,D). They were all cultured for up to 7 days with DMEM+10%FBS media to support proliferation and prevent differentiation. We also prepared a control device with cells cultured in 2D (on petri dish), which was different than the collagen I surface growth with much higher substrate stiffness. These cells grew to full confluency in 3 days with media (Figure 2B), and further growth was restricted in 2D at that point due to contact inhibition.

C2C12 cells cultured on the 2.5D soft substrate proliferated much faster than in 3D (Figure 2B), which was likely related to the cells on the surface having direct access to oxygen and nutrients. Furthermore, the collagen concentration was higher (3 mg/mL) for the 2.5D than the other 3D culture conditions (1–2 mg/mL) reported in literatures [32]; this concentration is the lowest limit for our microfluidic channel, while allowing us to maintain its structure and geometry. Cells cultured in 1 mm thick ECM system still proliferated at a moderate rate, but cells in the 2 mm ECM did not show any significant density change. These results could be observed quantitatively in Figure 2C. All of these experiments maintained a relatively high cell viability over 7 days indicating that cells in 3D (especially in the thicker ECM) likely transferred to a G_0_ phase in response to the more challenging environment [33]. But still, the eventual cell viability was higher on 2.5D surface than in 3D scaffold. In addition, cells in both 1 mm and 2 mm 3D conditions experienced a viability drop at Day 1, yet this drop was restored from Day 2 to Day 7 (Figure 2C). This response could be due to sudden nutrient insufficiency causing some of the C2C12 cells to die, yet other cells proliferated, which increased the viability over time.

The morphology of C2C12 in 3D when compared to 2.5D also showed that cells aligned locally in 2.5D, but not in 3D (Figure S1C,D From Day 4 to Day 7). This finding is commonly observed in 2D C2C12 culture when cell confluency is high [34]. As skeletal muscle cells, C2C12 differentiate and form myotubes with the alignment being a critical pre-indicator to myotube formation. However, this alignment did not occur in 3D, which suggests that myotube formation in our 3D design did not induce cell differentiation and cell fusing for myotube formation. Since only cells with high confluency could locally align with each other, C2C12 likely needed to remain in contact to sense each other to induce alignment. Thus, myogenesis may not have occurred in 3D due to low cell density.

**Figure 3:**
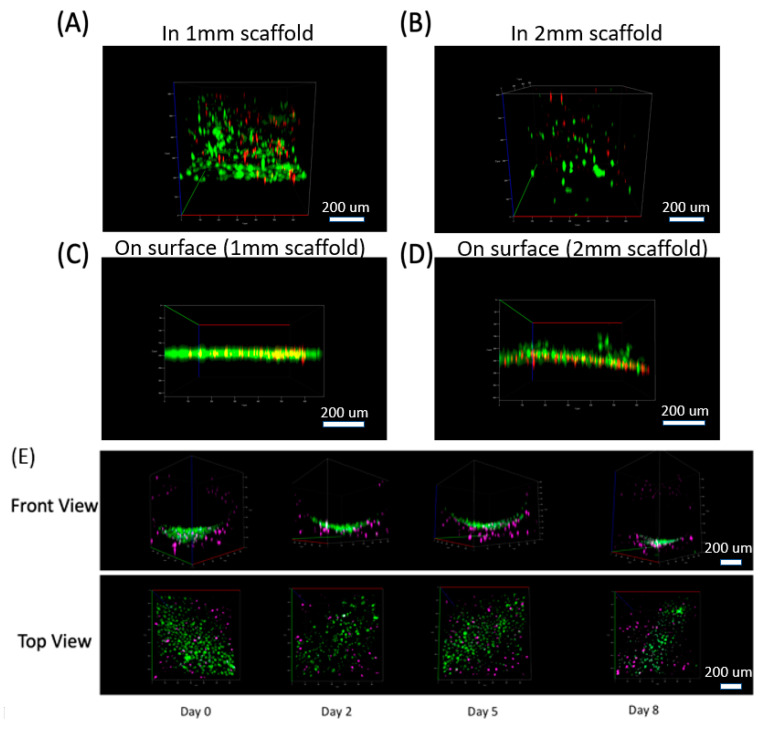
3D distribution of cells in our collagen scaffold. Cells distribution in 3D (front view) at Day 7 for 4 samples: (**A**) Cells seeded in 1 mm thick collagen scaffold, (**B**) 2 mm thick collagen scaffold, and cells cultured on the surface of (**C**) 1 mm thick collagen and (**D**) 2 mm thick collagen. (For A–D. Green: Live cells stained by Calcein AM. Red: Dead cells stained by ethidium homodimer). (**E**) Co-culture system for C2C12 cells in our 3D scaffold with HUVEC cells cultured in the channel (lower half of the channel shown here). HUVEC cells attached to the channel wall and formed a curved distribution along the circular channel wall. See Video S1 as well. (For E. Purple: C2C12 cells stained by Celltracker Deepred. Green: HUVEC cells stained by Celltracker Green).

We also fabricated microfluidic channels in the ECM through our micromilling approach [26,28]. Briefly, we developed a sacrificial gelatin template that was embedded in the ECM before collagen crosslinking, and then we removed it by increasing the temperature to 37 °C. We seeded HUVECs into the lower half of ECM microfluidic channels to mimic vasculature. To analyze this, we pre-stained C2C12 cells with CellTracker DeepRed and HUVEC cells with CellTracker Green for imaging. C2C12 cells were seeded into 1.5 mm thick scaffolding first. After crosslinking the collagen scaffold, we introduced DMEM media with dense HUVEC cells into the channel and incubated them in the channel for 2 h. The channel was imaged using a confocal microscope at regular intervals up to 8 days (Figure 3E). The HUVECs were attached to the channel wall and a single layer of cells was observed on the channel wall. 

### 3.2. Muscle Cell Differentiation and Myotube Formation

**Figure 4:**
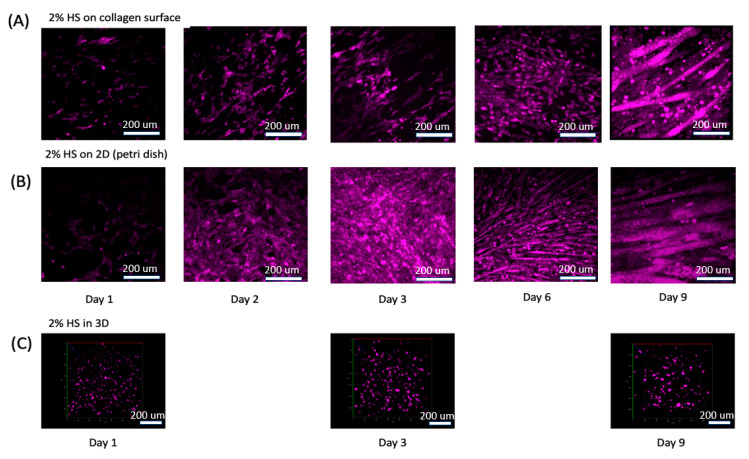
C2C12 were stained with CellTracker DeepRed and treated with DMEM+2%HS for up to 9 days. (**A**) C2C12s cultured on 2.5D collagen surface. (**B**) C2C12s cultured in a petri dish. (**C**) C2C12s cultured in our 3D ECM. See Videos S2–S4 for 3D rotational view of the cells.

We differentiated the C2C12s to form myotubes in order to generate muscle tissue mimetic systems. We applied DMEM with 2% horse serum (HS) to the C2C12s in 2D, 2.5D, and 3D as previously described in Figure S1. C2C12 cells in 2D and 2.5D conditions quickly aligned and displayed myogenesis. As C2C12 cell grew and differentiated in 2D and 2.5D, they merged into multi-nuclear myotubes and thus looks larger than single cells. As we checked the remaining single cells (at day 9), their size was the same as cells at day 1 and day 2 (Figure 4A,B). However, C2C12s in our 3D scaffold did not appear to change in density or in morphology (Figure 4C). We then increased the initial C2C12 culture density from 0.5 million/mL to 20 million/mL as we believed cell density in 3D could be part of this lack of response. We also thought nutrient supply, which is related directly to scaffold thickness, and collagen stiffness, and is related directly to collagen concentration, could limit cell differentiation and alignment ability, so we altered these as well (Figure S2). By reducing collagen density, the scaffolding had a visible remodeling effect. More specifically, the semi-transparent scaffold (Figure 2A) contracted significantly into less than half its initial size and became highly opaque even when visualizing with a confocal microscope (Figure S2, 2nd and 3rd row). With enough media supply, a thickness range of 0.5–2 mm did not significantly influence cell viability and growth. However, less thickness and lower collagen concentration both caused reduction of collagen scaffold strength and allowed C2C12 remodeling to occur more dramatically. Since vessel fabrication required a stable scaffold to maintain its morphology, we decided to set collagen fabrication thickness to 1.5 mm and collagen concentration to 3 mg/mL.

### 3.3. ECM Remodeling and New Channel Design

**Figure 5:**
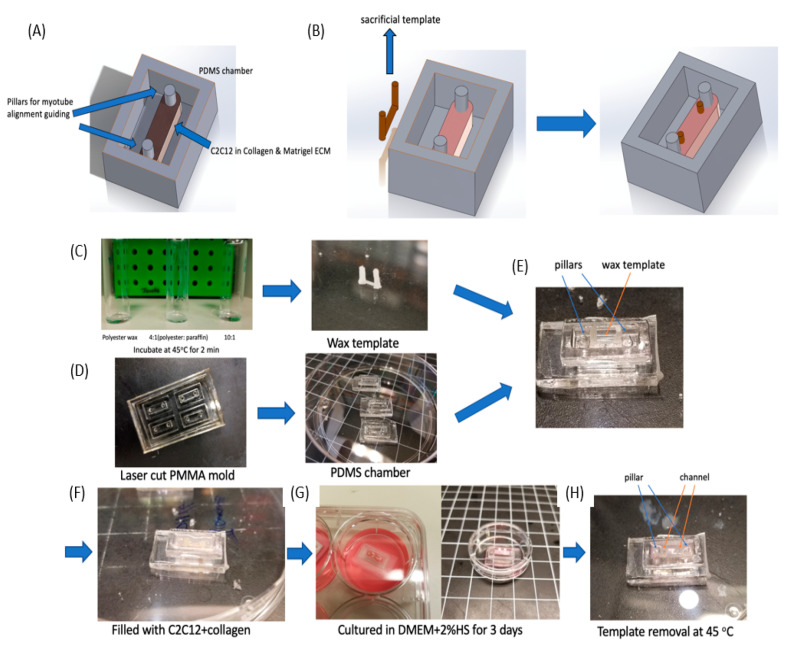
Chamber design for vascular conduits with the wax templates integrated with C2C12 differentiation. (**A**) A PDMS chamber (10 × 4 × 3 mm) was designed with two pillars to guide C2C12 cell alignment. (**B**) Design of the wax sacrificial template in the chamber. This sacrificial system is placed in the incubator for 2 days and is removed after myotube formation. (**C**–**H**) Fabrication process for a differentiated muscle tissue model with a built-in channel. (**C**) Wax is mixed with an appropriate polyester:paraffin ratio, which was selected to achieve a desired melting point. Then the wax template was fabricated through our molding method. (**D**) The PDMS chamber was fabricated from a laser cut PMMA mold. (**E**) The wax template was then placed in a PDMS chamber along the double-pillar direction. (**F**) Collagen was mixed with C2C12 cells and injected into the chamber to cover the wax template. (**G**) C2C12 was then cultured in DMEM+2%HS for 3 days to induce myogenesis. (**H**) The wax template was removed by heating the device to 45 °C quickly, which left a microfluidic channel behind.

C2C12 differentiation generally induces local myotube formation only, yet artificial muscle tissue generation is preferable if universal alignment can be induced. To guide myotube directionality, we applied two PDMS pillars in a chamber (Figure 5A). As C2C12s remodeled the ECM and caused contraction, the ECM wrapped around the pillars to generate parallel stresses, which then guided the myotubes to form in a parallel direction between the pillars.

Since one of our primary goals was to embed microfluidic channels into ECM scaffolds to mimic vessel system, we designed a sacrificial template (Figure 5B) and integrated it into the ECM scaffold. We expected to fabricate a single circular channel with an inlet and outlet and then planned to inject HUVEC cells into the channel and allow them to grow into monolayer. However, one major difference between synthetic muscle tissue fabrication and regular collagen embedded channel is ECM remodeling by C2C12 cells. As we observed in Figure S2, densely packed C2C12 cells remodeled the 3D collagen scaffold, usually causing contraction. This contraction would change the channel size and even collapse the channel during the 2–3 day myogenesis process. Therefore, instead of fabricating the channel immediately after collagen crosslinking, we kept the sacrificial template in the collagen for 3 days until myotubes were formed and the collagen remodeling was at an advanced state. Then by removing the sacrificial template at this point, we obtained an intact circular channel.

To accomplish this, we needed a different thermo-reversible material that would remain solid in a 37 °C incubator for 3 days. After myotube formation, we could then remove it by changing temperature again. A commonly utilized sacrificial template material such as gelatin [28] would be challenging as it would melt at 37 °C within a few minutes, and thus could not support the channel structure. Wax though can generally hold a very steady structure in solid form. Different wax types provide distinct melting points, but a melting point slightly above 37 °C was needed, so it could remain a solid in the incubator while being able to be easily removed with a slight increase in temperature. Thus, we applied a mixture of polyester wax (melting point = 37 °C) and paraffin wax (melting point ≈65 °C) to obtain this melting point by controlling the mixture ratios. To test the physical characteristics, we examined 3 mixture ratios: (1) Polyester wax only; (2) 4 (polyester wax): 1 (paraffin wax); and (3) 10 (polyester wax): 1 (paraffin wax) as shown in Figure 5C. All three samples were stored at 37 °C for 30 min, and then placed in a 45 °C oven for melting. The 10:1 ratio stayed in a solid form at 37 °C and then quickly melted at 45 °C. 

### 3.4. Differentiated Muscle Tissue and Vascular Mimetic System

For the fabrication of this muscle tissue model, we built off of our previous reported micro-milling and micro-molding method [28] to obtain a wax template with two cylinders that could be physically manipulated connected by a long circular channel (Figure 5C). Then we fabricated a PMMA mold to produced PDMS chamber (4 mm × 12 mm × 2.4 mm in size) with double pillar (diameter = 0.5 mm) structure (Figure S3 and Figure 5D). This PMMA mold was obtained by stacking 3 layers of PMMA together. By pouring liquid PDMS (10:1 ratio) into the mold and curing it at 60 °C for 2 h, we demolded the PDMS chambers. Then we carefully placed the wax template into the PDMS chamber and sterilized them in 70% ethanol for 2 h. We filled the chamber with C2C12 cells (Density = 3 × 10^7^ cells/mL) in liquid collagen (3 mg/mL) and incubated it at 37 °C for collagen crosslinking (Figure 5E,F).

**Figure 6:**
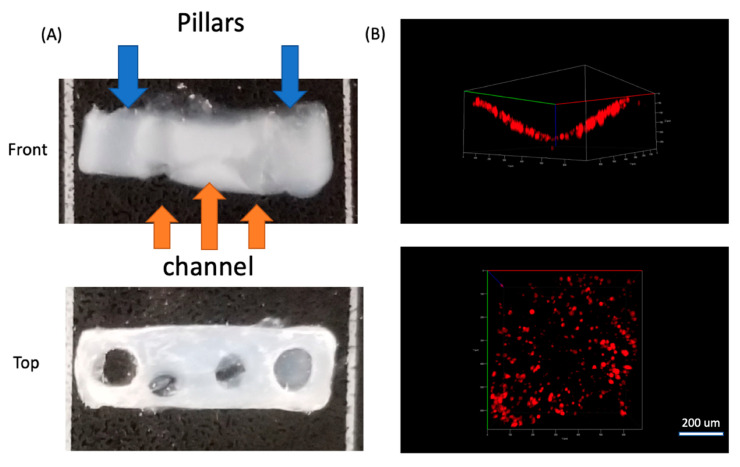
(**A**) Front and top view of the collagen scaffold with pillars and embedded channels. (**B**) Front and top view of HUVEC cells stained with CellTracker DeepRed cultured in the channel and imaged using confocal microscopy.

After the collagen crosslinking, we transferred the device into a 6-well plate and immersed it in DMEM+2%HS for 3 days, so that the C2C12 cells could differentiate to form myotubes between the two pillars (Figure 5G). During this period the ECM contracted and generated tension between the pillars, while the wax template remained solid. After ECM contraction, the device was moved to an oven at 45 °C, and the wax quickly melted and was removed with tweezers and a syringe. PBS was injected twice into the channel to remove remaining wax residue, which resulted in a circular channel embedded in an ECM scaffold (Figure 5H and Figure 6). Figure 6B showed the side and top view of HUVEC cells cultured in half of the channel. The side view showed the geometry of channel, which confirmed single layer distribution of HUVEC cells, and the top view presented a uniform distribution of the cells in the channel. Since we only allowed the sample stayed in the oven for 2 min, which will not raise the temperature of the sample to 45 °C. We carefully designed the wax ratio to control its melting temperature, it should only be slightly above 37 °C. Therefore, the cells experienced slightly higher than 37 °C for 2 min. This result is also reconfirmed by a viability test after the treatment (Figure S4).

In this approach with the double-pillar guides, cells in 3D appeared to extend and connect head to tail, which was a sign of myotube formation. Between the two pillars, cells mainly aligned horizontally (Figure S4A,C), yet for cells on both edges, they aligned vertically (Figure S4B). Their alignment seemed to match the local stress direction and was likely caused by tension generation. Live/dead staining indicated that there were mainly living (green) cells (Figure S4A–C). We then seeded HUVEC cells into the channel (lower half) to form a monolayer on the inner circular channel wall. We first stained HUVEC cells with CellTracker DeepRed, and then introduced the cells with the culture media into channels and incubated them for 2 h. Then the media was removed, and PBS was introduced twice to remove any unattached cells. Confocal images of HUVEC cells (Figure 6B) showed HUVECs attached to the region of the channel wall uniformly in a monolayer where they were cultured. 

For in vitro tissue-on-a-chip systems, skeletal muscle tissues are challenging due to myogenesis with significant ECM remodeling effects [35]. The biological response generally induces ECM contraction, and even can create contractile pulses of the entire tissue with or without electrical stimulation [36]. Thus, the mechanobiology and geometric features of skeletal muscle tissues play a critical role for muscle-on-a-chip development. Recently, approaches were specifically designed to control the geometric and spatial forms of skeletal muscle tissues [37], such as 3D bio-printed C2C12 myotubes [38], and C2C12 cultured on micropatterned substrates [39].

Our design provides an alternative approach to fabricate 3D muscle-on-a-chip system with vasculature, by adapting sacrificial templates with unique thermo-responsive materials. The wax template with controlled thermal responses allowed the tissue system to respond better to the mechanical influence with myogenesis, and thus enabled the geometric features to be integrated into the muscle-on-a-chip system. 

## 4. Conclusions

We successfully developed a 3D myogenesis approach with a circular microfluidic channel embedded in an ECM scaffolding. C2C12 cell differentiation and myotube formation in 3D collagen scaffold were achieved with horse serum-based media, and myotube generation was guided with a double-pillar setup using a PDMS chamber. We used micro-milling and micro-molding with themoresponsive polymers to create this muscle-based vessel embedded system. We created a vessel using a polyester wax and paraffin wax mixture for the sacrificial template to generate an ECM embedded microfluidic channel. We also successfully seeded HUVECs in the ECM microfluidic channels to mimic the vessel system. In the future, generating more complex vascular mimetic channels including branched networks would allow for even more nutrient and oxygen transport and coverage within these ECM scaffoldings. This work will have impact in a diversity of directions including tissue-on-a-chip system, drug discovery approaches, and personalized tissue transplantation.

## Supplementary Materials

The following are available online at https://www.mdpi.com/2072-666X/11/10/907/s1, Figure S1: C2C12 cells cultured in the microfluidic channel ECM scaffolding system for up to 7 days. Figure S2: C2C12 cells cultured in a 3D scaffold for 6 days with DMEM+2%HS. Figure S3: Schematic of the fabrication of the PDMS chamber for ECM embedding. Figure S4: Cell alignment in the ECM without the channel. Video S1: Rotational view of Figure 3E. Co-culture system for C2C12 cells in 3D scaffold with HUVEC cells cultured in the channel (lower half of the channel). HUVEC cells attached to the channel wall and formed a curved distribution along the circular channel wall. Video S2: 3D rotational view of C2C12 cells in 3D at day 1. Video S3: 3D rotational view of C2C12 cells in 3D at day 3. Video S4: 3D rotational view of C2C12 cells in 3D at day 9.
