# Supplementary material for: Toward Vasculature in Skeletal Muscle-on-a-Chip through Thermo-Responsive Sacrificial Templates
Source: Micromachines (Basel). 2020 Sep 30;11(10):907. doi: 10.3390/mi11100907 (PMC7601354; doi:10.3390/mi11100907)
Supplement: Supplementary file 1 [file micromachines-11-00907-s001.zip › micromachines-941590-si proofed/supplement.pdf]

Supplementary Materials

Toward vasculature in skeletal muscle-on-a-chip through themoresponsive sacrificial templates

Li Wan[1], James Flegle[2], Burak Ozdoganlar[1], Philip R. LeDuc[1]

[1] Department of Mechanical Engineering, Carnegie Mellon University, 5000 Forbes Ave, Pittsburgh, PA 15213, USA.
[2] Department of microbiology, University of Chicago, 5801 S Ellis Ave, Chicago, IL 60637, USA.

* Correspondence: prl@andrew.cmu.edu

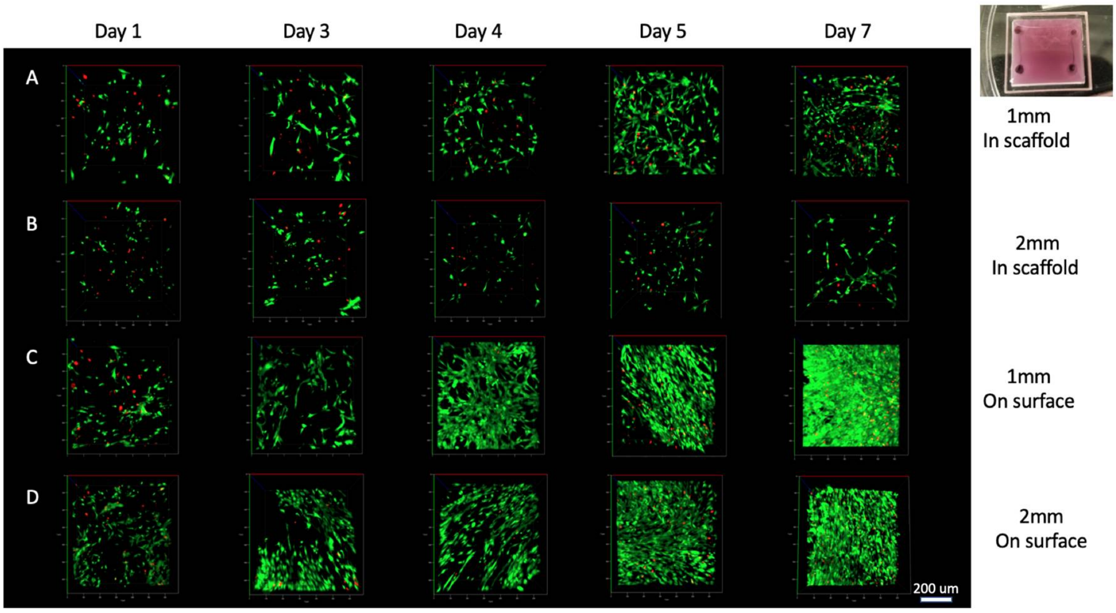

Figure S1. C2C12 cells cultured in the microfluidic channel ECM scaffolding system for up to 7 days. (A) Cells were seeded in the 1mm thick collagen scaffold. (B) Cells were seeded in the 2mm thick collagen scaffold. (C) Cells were cultured on the surface of the 1mm thick collagen. (D) Cells were cultured on the surface of the 2mm thick collagen. The green stain is CalAM, which labels for live cells, and the red stain is EthD-1, which labels for dead cells.

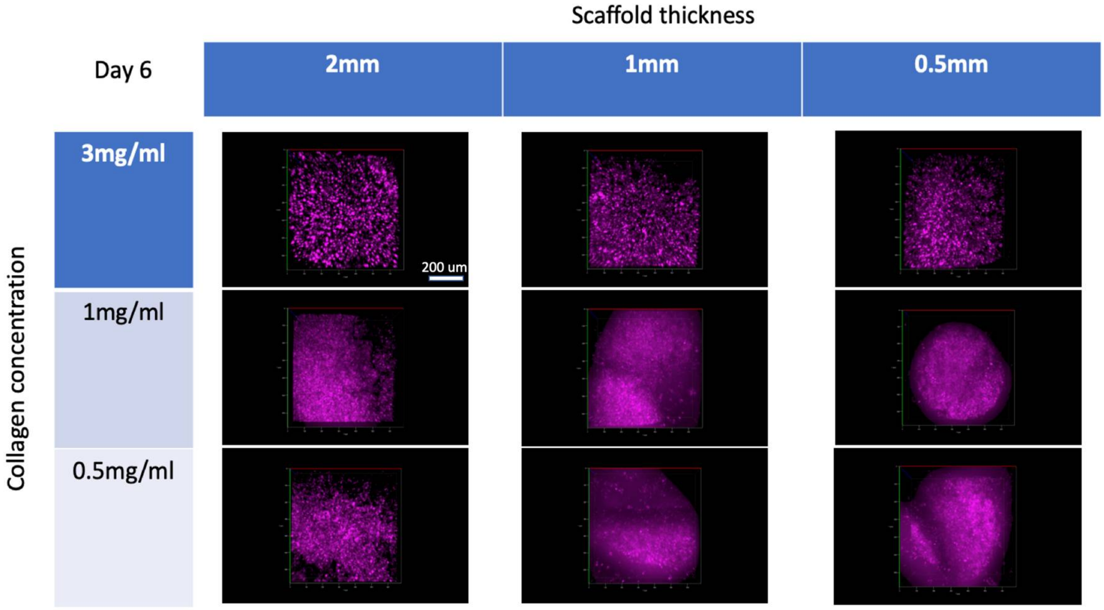

Figure S2. C2C12 cells cultured in a 3D scaffold for 6 days with DMEM+2%HS. Cell density = 20 million/ml. We utilized multiple scaffold thicknesses (0.5mm-2mm) and collagen concentrations (0.5mg/ml-3mg/ml). For cells with low collagen concentration, collagen remodeling occurred along with scaffold contraction.

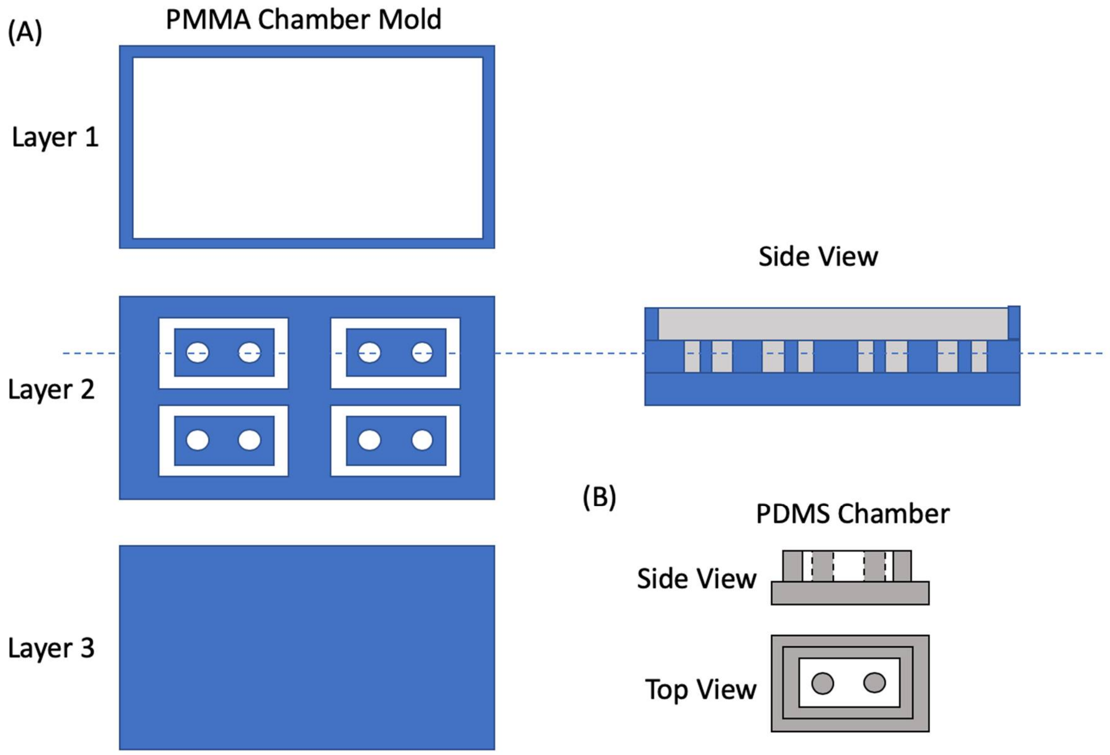

Figure S3. Schematic of the fabrication of the PDMS chamber for ECM embedding. (A) A PMMA chamber mold was created by laser cutting 3 layers of PMMA (2.4mm in thickness) and adhered with UV sensitive glue (NOA 81). Then the PDMS (10:1) was poured into the PMMA mold and incubated at 60 ^{o}C for 2 hours. (B) The PDMS was then detached from the PMMA mold and cut into single chambers.

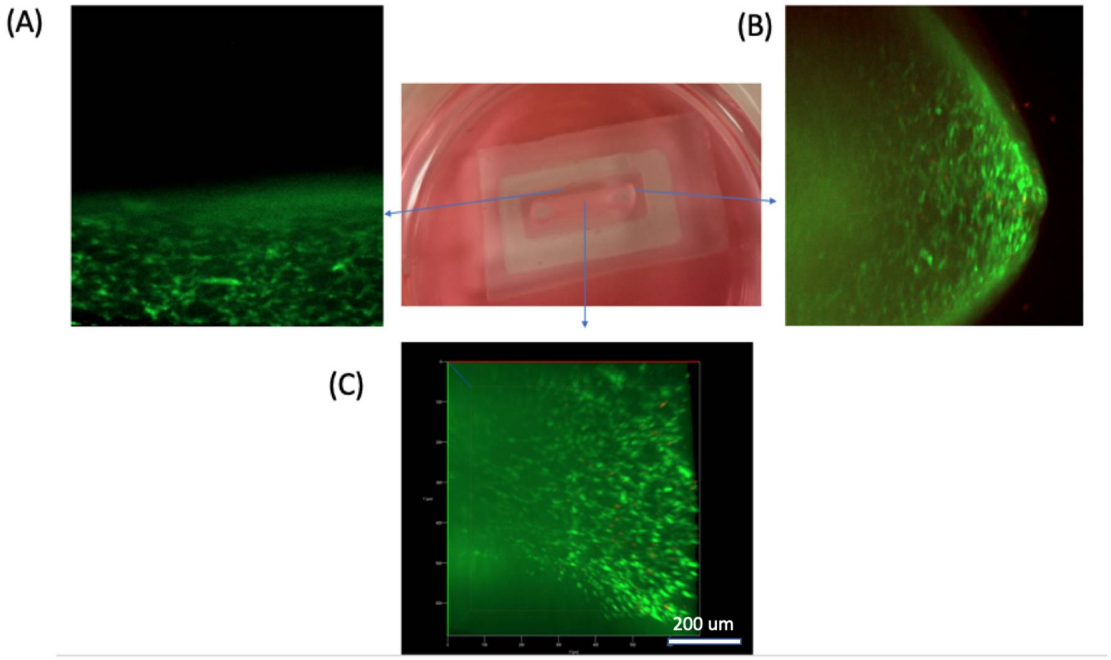

Figure S4. Cell alignment in the ECM without the channel. C2C12 cells aligned along self-generated tension regions of ECM. This was mostly horizontal for cells between the two pillars (A&B), and vertical for cells on edge (C). Live/dead staining (Green: live/Red: dead) revealed high cell viability in the system.

Movie 1. Rotational view of Figure 3E. Co-culture system for C2C12 cells in 3D scaffold with HUVEC cells cultured in the channel (lower half of the channel). HUVEC cells attached to the channel wall and formed a curved distribution along the circular channel wall.

Movie 2-4. Rotational view of Figure 4C. C2C12s cultured in our 3D ECM.

Movie 2. 3D rotational view of C2C12 cells in 3D at day 1.

Movie 3. 3D rotational view of C2C12 cells in 3D at day 3.

Movie 4. 3D rotational view of C2C12 cells in 3D at day 9.
